# The Association Between Physical Activity, APOE Genotype, and Cognitive Performance in Cognitively Healthy Older Adults: Findings from a Brazilian Cohort

**DOI:** 10.1002/alz70855_106880

**Published:** 2025-12-24

**Authors:** Rebeca Carvalho Bom, Leticia Fernanda Palma, Karen Leticia Pulgatti, Vanessa Alexandre‐Silva, Sabrina Dorta de Oliveira, Cecilia Patricia Popolin, Marcia Regina Cominetti, Lucas Nogueira de Carvalho Pelegrini

**Affiliations:** ^1^ Federal University of Sao Carlos, São Carlos, Sao Carlos, Brazil; ^2^ Federal University of São Carlos, São Carlos, SP, Brazil; ^3^ Federal University of Sao Carlos, Sao Carlos, SP, Brazil; ^4^ Federal University of São Carlos, São Carlos, São Paulo, Brazil; ^5^ The Global Brain Health Institute, Trinity College Dublin, Dublin, Dublin, Ireland

## Abstract

**Background:**

Population aging leads to changes that impact cognition, with advanced age being a major risk factor for neurocognitive disorders. The presence of the APOE ε4 allele is one of the strongest genetic risk factors for Alzheimer's disease, while non‐pharmacological interventions, such as physical activity (PA), have been linked to better cognitive outcomes. Thus, the present study aims to verify whether physical activity is associated with cognition in cognitively healthy older adults, considering their APOE status.

**Method:**

This cross‐sectional, observational study examined whether PA is associated with cognitive performance in cognitively healthy Brazilian older adults, considering their APOE genotype. A total of 83 participants were allocated into APOE ε4 carriers (ε4+) and non‐carriers (ε4−). The assessment consisted of a sociodemographic questionnaire and the Addenbrooke's Cognitive Examination‐Revised (ACE‐R). Blood samples were collected from participants on a previously scheduled day. APOE genotyping was determined via qPCR, and statistical analyses included descriptive statistics, group comparisons, correlation analyses, and ANCOVA (*p* <0.05). Analyses were conducted on SPSS 29.0, and the significance level was set at *p* <0.05. All participants signed informed consent forms prior to data acquisition.

**Results:**

Of the sample, 26% were APOE ε4+, 62.7% were female, 73.5% identified as white, and 71.1% reported engaging in PA (mean duration = 11.2 years). While ε4 carriers exhibited lower cognitive performance, no statistically significant differences in cognition or sociodemographic variables were found between groups. Correlation analyses confirmed the expected relationship between ACE‐R scores, age, and education but revealed no significant relationship between global cognition and PA or APOE status. ANCOVA showed that APOE status significantly influenced cognitive performance [F(1,48) = 4.51; *p* = 0.039], whereas PA had no independent effect after controlling for APOE status [F(23,43) = 0.86; *p* = 0.644].

**Conclusion:**

These findings suggest that APOE ε4 carrier status impacts cognitive performance of cognitively unimpaired older adults, but PA was not independently associated with cognition in this sample. While PA is known to benefit brain health, its direct cognitive effects may be influenced by genetic factors, highlighting the need for larger, longitudinal studies to further investigate these relationships.

